# Activity Measure for Postacute Care to Predict Discharge Destination After Surgery

**DOI:** 10.1002/wjs.70203

**Published:** 2025-12-17

**Authors:** Daniel S. Rubin, Rachel Baccile, Abraham Trachtman, Ryan P. Merkow, Maylyn Martinez

**Affiliations:** ^1^ Department of Anesthesia and Critical Care University of Chicago Chicago Illinois USA; ^2^ The Center for Health and the Social Sciences University of Chicago Chicago Illinois USA; ^3^ Northeastern Illinois University Chicago Illinois USA; ^4^ Center for Surgical Implementation and Health Services Research (SIHSR), Department of Surgery University of Chicago Chicago Illinois USA; ^5^ Section of Hospital Medicine, Department of Medicine University of Chicago Chicago Illinois USA

**Keywords:** abdominal surgery, discharge, mobility, postacute care, risk prediction model

## Abstract

Area under the receiver operating characteristic curve for the Activity Measure for Post‐Acute Care (AM‐PAC) model and American College of Surgeons National Surgical Quality Improvement Program (NSQIP) risk calculator model to predict discharge to postacute care.

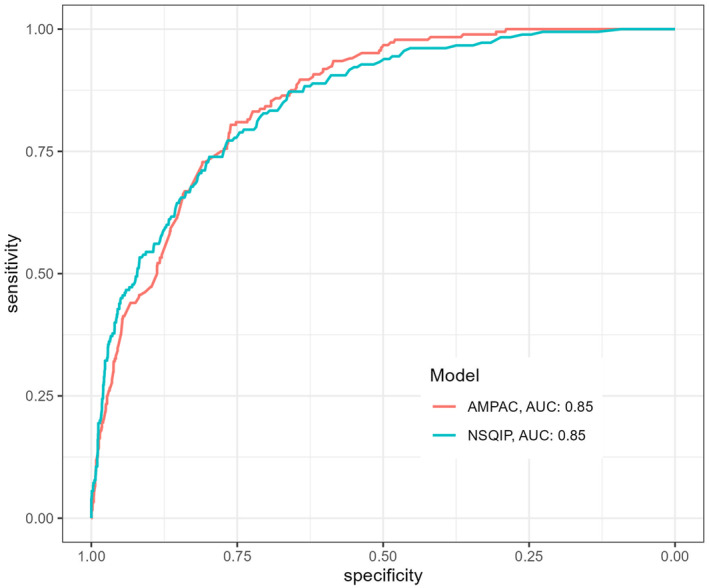

## 1 Introduction

Postoperative discharge destination is a key patient‐centered outcome [1]. Identifying patients at risk for discharge to a postacute care (PAC) facility (e.g., skilled nursing facility) can help triage interventions designed to prevent functional decline and facilitate early discharge planning [2]. The American College of Surgeons National Surgical Quality Improvement Program (ACS NSQIP) developed a machine learning algorithm to predict discharge to PAC. However, widespread implementation of this risk calculator remains limited, as the calculator requires manual entry of 21 independent variables and cannot be integrated into hospitals’ electronic medical records (EMR) due to its proprietary algorithm [3]. Functional status, particularly as measured by activities of daily living, is a well‐established determinant of discharge disposition [4]. The Activity Measure for Post‐Acute Care (AM‐PAC) Inpatient Mobility Short Form is a validated tool that assesses difficulty and need for assistance with six routine mobility tasks (i.e., “turning over in bed”) and is already widely collected in hospitals. Prior work in medical inpatients has shown that AM‐PAC scores, when combined with routine clinical characteristics, demonstrate robust prediction of discharge to PAC, with the AM‐PAC score as the most important predictor [5]. In this study, we evaluated the use of admission AM‐PAC scores combined with routinely collected clinical variables to develop a discharge prediction model in surgical patients and compared its performance to the ACS NSQIP discharge to PAC risk calculator.

## 2 Methods

We conducted a single‐center retrospective cohort study from June 1, 2016, to May 31, 2023, of patients undergoing major abdominal surgery. This study was approved by the University of Chicago Institutional Review Board (IRB), and the requirement for written informed consent was waived by the IRB. We used data from the ACS NSQIP Participant Use Data Files and the EMR at a large tertiary urban academic medical center. Patients were admitted to general, gynecology, urology, or colorectal surgery services. Admission AM‐PAC scores, collected as part of routine clinical care, were extracted from the EMR. Patients were excluded if data were missing on elective or emergent surgery classification (*n* = 710), were admitted from a skilled nursing facility (*n* = 96), underwent a nonabdominal primary procedure (*n* = 51), lacked a primary current procedural terminology (CPT) code (*n* = 23), were discharged to hospice (*n* = 9), or were missing an admission AM‐PAC score (*n* = 1), yielding a total of 806 exclusions. The primary outcome was discharge to PAC. Covariates included age (18–49, 50–64, over 65), sex, body mass index, number of comorbidities, admission AM‐PAC score, origin (home vs. acute care hospital), American Society of Anesthesiologists Physical Status Classification System (ASA‐PS) score, operative stress score (OSS as derived by the primary CPT code), and elective versus nonelective surgery. We used multivariable logistic regression to estimate the association between covariates and odds of discharge to PAC. For comparison, ACS NSQIP data were manually entered into the ACS NSQIP risk calculator to generate the predicted risk of discharge to PAC. Model performance was evaluated using the area under the receiver operating characteristic curve (AUC) to compare the AM‐PAC‐based model with the NSQIP model. All analyses were performed using Stata version 18 (StataCorp, College Station, TX).

## 3 Results

**FIGURE 1:**
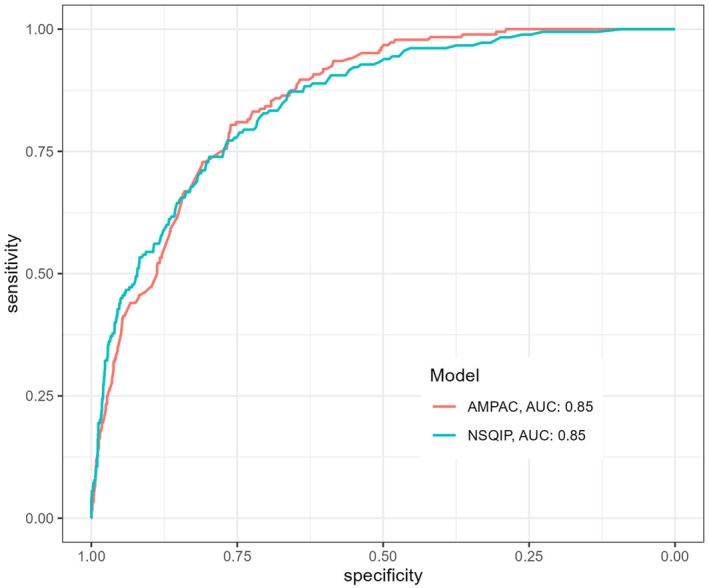
Area under the receiver operating characteristic curve for the Activity Measure for Post‐Acute Care (AM‐PAC) model and American College of Surgeons National Surgical Quality Improvement Program (NSQIP) risk calculator model to predict discharge to postacute care.

We included 2448 hospitalized surgical patients, of whom 196 (8.0%) were discharged to PAC. The median age was 59 (IQR: 44–70) years, 51.5% were female, and the median AM‐PAC score at admission was 18 (IQR: 16–19). As shown in Table 1, factors independently associated with increased odds of discharge to PAC included older age, having two or more comorbidities, nonelective surgery, an ASA score of 3 or 4, an OSS of 4 or 5, and an admission AM‐PAC of ≤ 18. The AM‐PAC‐based prediction model demonstrated high classification accuracy with an AUC of 0.85 (95% CI: 0.83, 0.88). The ACS NSQIP risk calculator showed a similarly high AUC of 0.85 (95% CI: 0.82, 0.88). There was no difference in model performance between the AM‐PAC and ACS NSQIP models (*p* = 0.86) (Figure 1).

**TABLE 1:** Patient characteristics and model results.

	*N* (%)	Outcome: discharge disposition to PAC	
		OR	95% CI
Total	2448		
Demographics			
Age category			
18–49	825 (33.7)	(ref)	
50–64	704 (28.8)	2.55	(1.29, 5.05)
65+	919 (37.5)	8.23	(4.42, 15.33)
Male	1187 (48.5)	0.87	(0.60, 1.19)
Body mass index (kg/m^2^)			
Normal (≥ 18.5 & < 25)	853 (34.8)	(ref)	
Underweight (< 18.5)	126 (5.2)	0.89	(0.43, 1.90)
Overweight (≥ 25 & < 30)	727 (29.7)	0.79	(0.51, 1.17)
Obese (≥ 30)	742 (30.3)	0.79	(0.53, 1.23)
Comorbidities			
Count of comorbidities			
0	1485 (60.7)	(ref)	
1	714 (29.2)	1.50	(1.03, 2.19)
2+	256 (10.2)	2.44	(1.55, 3.84)
Mobility			
Admission activity measure postacute care > 18	763 (31.2)	0.28	(0.17, 0.46)
Admission details			
Origin			
Home	2353 (96.1)	(ref)	
Acute care facility	95 (3.9)	1.67	(0.84, 3.31)
Surgery details			
ASA category			
1 or 2	888 (36.3)	(ref)	
3 or 4	1560 (63.7)	2.78	(1.59, 4.84)
Elective	2086 (84.8)	0.13	(0.08, 0.21)
Operative stress score			
2 or 3	776 (31.7)	(ref)	
4 or 5	1672 (68.3)	2.85	(1.78, 4.57)

## 4 Discussion

An admission AM‐PAC score combined with seven routinely collected clinical variables was as accurate as the updated ACS NSQIP risk calculator in identifying major abdominal surgical patients at risk for discharge to a PAC facility. A key advantage of our model is the ability to integrate it into any hospital’s EMR to make these results readily available to clinicians to identify at‐risk patients on the first day of hospitalization. Implementing this tool at admission may help to set patient and family expectations, facilitate early discharge planning, and triage inpatient services such as physical therapy to patients at the highest risk of discharge to PAC. Our study has limitations, including its single‐center design, which may limit its generalizability. Future research is warranted to validate this model in other hospital systems and patient populations. Finally, this risk score could be evaluated as a tool to identify high‐risk patients for targeted interventions to reduce the frequency of discharge to PAC.

## Author Contributions

**Daniel S. Rubin:** conceptualization, data curation, formal analysis, funding acquisition, investigation, methodology, project administration, resources, software, supervision, validation, writing – original draft, writing – review and editing. **Rachel Baccile:** data curation, formal analysis, investigation, software, writing – review and editing. **Abraham Trachtman:** data curation, investigation, writing – review and editing. **Ryan P. Merkow:** investigation, validation, writing – review and editing. **Maylyn Martinez:** conceptualization, funding acquisition, investigation, project administration, supervision, writing – original draft, writing – review and editing.

## Funding

This study was supported by the National Institute on Aging (R03AG078957) and the National Institute on Minority Health and Health Disparities (K23MD019072).

## Conflicts of Interest

The authors declare no conflicts of interest.
